# Mixed Adeno-Neuroendocrine Carcinoma; Case Series of Ten Patients with Review of the Literature

**DOI:** 10.4274/balkanmedj.2017.1471

**Published:** 2018-05-29

**Authors:** Yiğit Düzköylü, Orhan Aras, Erdal Birol Bostancı, Tülay Keklik Temuçin, Murat Ulaş

**Affiliations:** 1 Clinic of Gastroenterological Surgery, Türkiye Yüksek İhtisas Training and Research Hospital, Ankara, Turkey; 2 Clinic of Pathology, Türkiye Yüksek İhtisas Training and Research Hospital, Ankara, Turkey

**Keywords:** Digestive system, mixed adenoneuroendocrine carcinoma, neuroendocrine neoplasm, prognosis

## Abstract

**Aims:**

Mixed adeno-neuroendocrine carcinoma is a rare entity, diagnosed with immunohistochemical studies. Literature mainly includes case reports and series which are very few. In our study, we aimed to report a case series from a tertiary hospital with demographics of the patients, detailed tumor and clinical findings and follow-up plus survival conditions.

**Methods:**

Pathology database was explored for patients with the pathological diagnosis of ‘mixed adeno-neuroendocrine carcinoma’ and patients were identified retrospectively and evaluated in means of demographics, histopathological examination, tumor properties.

**Results:**

Ten patients had been diagnosed with mixed adeno-neuroendocrine carcinoma in our center, diagnosed at a mean age of 64.7. Stomach was found to be the most common localization. Five patients (50%) were diagnosed as grade 3. Following surgery, median follow-up was 15 months with a median survival time of 20.6 months.

**Conclusion:**

This case series may contribute to the literature on the pathological and clinical aspects of the mixed adenoneuroendocrine carcinoma of the gastrointestinal system.

Mixed adenoneuroendocrine carcinoma (MANEC) is a very rare entity and has been defined in 2010 by the World Health Organization (WHO) as neoplasms involving both the epithelial and the neuroendocrine components. Two of the most important diagnostic criteria are (i) each component has to comprise at least 30% of the tumor and (ii) both components must be malignant (1). Cordier was the first researcher to describe a gastrointestinal tumor involving both the neuroendocrine and the exocrine components in 1924 (2). Later, Lewin (3) published the first systematic classification of mixed tumors with adenocarcinoma and neuroendocrine components and divided these tumors into three different groups, including combined, collision, and amphicrine tumors, in 1987. Following the first histopathological classification, La Rosa et al. (4) described these tumors classifying them as high-grade and intermediate-grade malignant tumors. The latest recommendation of the WHO suggests that each histologic component should be separately graded and evaluated (1,5). Till date, in the literature, MANECs have been identified in various organs such as the stomach (6), the colon (7), the biliary tract (8), the pancreas, and even the uterine cervix (9). MANECs are very rare tumors, and biliary localization is highly exceptional. In our case series, these tumors were diagnosed as follows: one in the pancreas, one in the ampulla, and one in the choledochus. Prior to our study, we found only 22 cases of ampullary MANECs in the literature (10). The latest WHO recommendations suggest that MANECs should be treated as adenocarcinoma; however, up-to-date evidence according to numerous authors indicates that the treatment should be based on the most aggressive histologic component (4,5,11). In addition, Chen et al. (12) have demonstrated high volume of high-grade NET component (>50% of the total tumoral volume) as an independent poor prognostic factor in gastrointestinal MANECs. High-grade MANECs usually exhibit higher values of Ki-67 proliferation index, which results in worse survival rates (13). Conversely, in lower grades of MANECs, the exocrine component of the tumor has been shown to be more aggressive than the neuroendocrine component (14). Because of the rarity of these tumors, a significant debate still exists regarding the treatment of MANECs. These tumors exhibit neither specific symptoms nor specific radiological or laboratory findings; thus, the diagnosis depends on postoperative histopathological and immunohistochemical studies. Although most of the case studies published in the literature so far have reported about treatment with surgery alone, few researchers also suggest treatment with somatostatin analogs in the presence of somatostatin receptor positivity (5,15). Published literature concerning MANECs is still limited and primarily based on single case reports. Diagnosis, surgical management, and follow-up criteria about these tumors are not yet clear. In our case series, we describe about 10 cases of MANECs from a tertiary center, which, according to our knowledge, is not only one of the largest case series from a single center but also the first extended report from a Turkish population. 

## MATERIALS AND METHODS

Following the approval of the local ethics committee, the pathology database was explored to identify patients with a pathological diagnosis of “MANEC” at a single tertiary referral center. The diagnosis had been made based on the current WHO classification, defining that each tumor component had at least 30% of the specimen. The patients were identified retrospectively and evaluated for their demographic details, histopathological examination results, and tumor properties, including immunohistochemical markers, stage at diagnosis, treatment modalities, clinical outcome, and survival follow-up. The mean follow-up duration was 18.1 months (minimum: 3, maximum: 51 months) starting from the date of operation. 

## RESULTS

**Figure 1:**
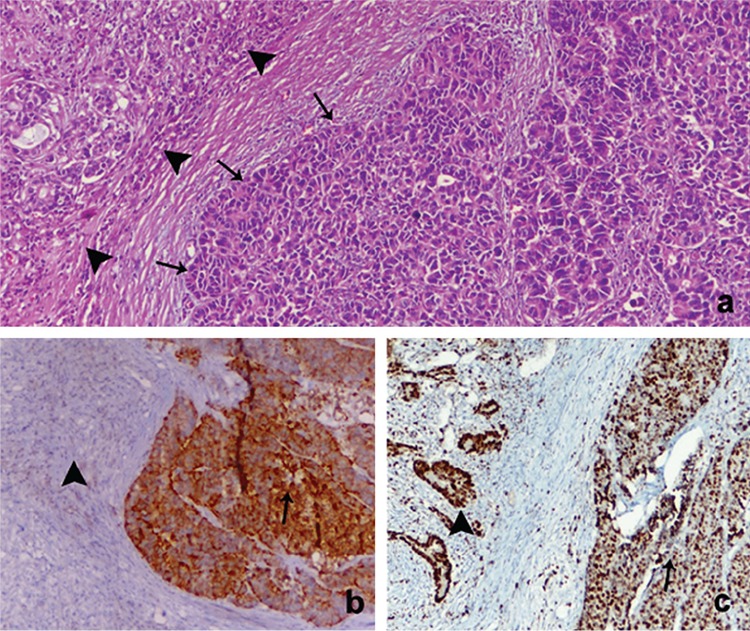
Histopathology and immunohistochemistry of mixed adenoneuroendocrine carcinoma. Mixed adenoneuroendocrine carcinoma of the stomach; H&E, x40 (a), Neuroendocrine component showing positivity for synaptophysin, x20 (b) and high Ki-67 labeling index in both components, Ki-67 x20 (c).

A total of 10 patients had been diagnosed with MANECs in our center. The demographic details of the patients and other evaluation criteria are shown in Table 1. Nine patients were males and one patient was a female. The mean age at diagnosis was 64.7 years (range: 48-73 years). All the patients had undergone surgery. The histopathological examination confirmed that all the patients had been treated with curative surgery (R0 resections, tumor-free resection margins). Distant liver metastasis was observed in only one patient. The dominant component was an adenocarcinoma in four patients and a neuroendocrine tumor in six patients. The most common tumor localization was the stomach (five patients). Other tumor sites included the rectum, the pancreas, the ampulla of Vater, the sigmoid colon, and the ductus choledochus. The adenocarcinoma component was well-differentiated in six patients, medium-differentiated in three patients, and low-differentiated in one patient. The grades of the neuroendocrine component were as follows: grade 1 in three patients, grade 2 in one patient, grades 2-3 in one patient, and grade 3 in five patients. The histopathological images and the immunohistochemistry results of a gastric MANEC are shown in Figure 1b, 1c. Surgery alone had been performed in five patients, while adjuvant chemotherapy was administered to the remaining five patients. None of the patients had been operated for a recurrent disease. Following surgery, the median follow-up duration was 15 months (range: 3-51 months). At the time of the study, six patients were alive with a mean survival time of 23.5 months (range: 10-51 months). Four patients had died with a median survival time of 20.6 months (range: 3-22 months). Patient no. 3 had died due to acute exacerbation of chronic obstructive pulmonary disease in the postoperative third month. Patient no. 4 had died in the 22^nd^ month due to irresectable local recurrence of gastric cancer. Patient no. 6 had died due to acute myocardial infarction during adjuvant chemotherapy in the third month. Patient no. 8 had died following the occurrence of multiple liver metastasis during the early postoperative term in the fifth month during neoadjuvant therapy.

## DISCUSSION

MANEC is a rare entity that has been defined differently since its first definition by Cordier in 1924 and several years later by Lewin in 1987 until the final description given in 2010 by the WHO as neoplasms involving both the epithelial and the neuroendocrine components. Because of the rarity of the tumor, the diagnostic criteria and the treatment options have differed according to different researchers. Latest developments in immunohistochemical staining techniques have led to an increased frequency of identifying few neuroendocrine cells in adenocarcinomas in the tumor tissues. Similarly, exocrine components can be commonly found in especially high-grade gastrointestinal neuroendocrine carcinomas (NECs) (4). However, the latest MANEC definition of the WHO necessitates the presence of each component comprising at least 30% of the tumor and both components being malignant (1). Due to the lack of any specific radiologic or symptomatic predictors of the tumor, the diagnosis is made based on the histopathological findings. Although the etiopathogenesis of the tumor is completely unclear, immunohistochemical studies have confirmed that the NEC component may give rise to the adenocarcinoma component, with both of them deriving from a single stem cell (16). Till date, in the published literature, MANECs have been identified in various localizations, with the colon (7) and the stomach (6) being the most common. Biliary and pancreatic MANECs are highly exceptional entities, and prior to our study, 22 cases of ampullary MANECs have been reported in the literature (10). In our study, we diagnosed one tumor in the pancreas, one in the ampulla, and one in the choledochus. We had performed cholecystectomy and hepaticojejunostomy following the excision of the extrahepatic biliary tree with tumor-free margins. Other localizations were gastric tumor in five patients, one in the rectum, and one in the sigmoid colon. The tumor was also diagnosed in the uterine cervix (9). The presence of MANECs has been reported predominantly in females due to a limited number of case series published in the literature (5,14). Conversely, a higher ratio of male patients was observed in our case series (9/10 patients, 90%). La Rosa et al. (17) have shown that the majority of MANEC tumors (>50%) stain positively for both chromogranin and synaptophysin. Similarly, in our study, positivity for these two protein markers was observed in 60% of the cases (six patients). In our case series, the rate of lymphatic invasion was found to be similar to that reported in previous series (50%), whereas the perineural invasion rate was lower (20%) (4,14). Harada et al. (5) defined six biliary MANEC cases with both perineural and vascular invasions (5). Similarly, in our case series, we identified both invasions in our patient with the choledochal MANEC, while the patient with the ampullary MANEC showed positivity only for lymphovascular invasion. The rarity of the tumor has resulted in an uncertainty in determining the optimal treatment strategy till date (18). The majority of researchers recommend considering the more aggressive component of MANEC (19), although few studies indicate that MANEC with a well-differentiated neuroendocrine component (grade 1 or 2) should be treated as an adenocarcinoma, whereas MANECs with grade 3 NEC should be treated as an NEC (4,20). Treating according to the more aggressive component of the tumor appears to be reasonable considering the fact that the aggressiveness of the neuroendocrine component of MANEC is based on the mitotic index and the Ki-67 proliferation index (3). As a result, although the latest WHO report suggests that these tumors be treated as adenocarcinomas, the latest evidence indicates that the treatment should be based on the most aggressive histologic component (4,5,11). In our case series, the NEC was the dominant component in 60% of patients. The NEC was grade 1 in three patients, grade 2 in one patient, grades 2-3 in one patient, and grade 3 in five patients. Among the 10 patients, the adenocarcinoma component was low-differentiated in only one patient. None of our patients had received neoadjuvant therapy, and curative surgery had been performed in all patients, with tumor-free margins in 100%. MANECs are rare tumors, and the majority of cases have been diagnosed with distant metastases in the literature (12). Conversely, only one of our patients with a sigmoid colon tumor had liver metastasis. In the literature, periampullary MANECs have been suggested to be treated surgically like other periampullary tumors, with pancreaticoduodenectomy being performed whenever possible (10). In our patient with the MANEC in the ampulla of Vater, a successful pancreaticoduodenectomy had been performed, and distal pancreatectomy had been performed in another patient with pancreatic MANEC. 

MANECs have been reported to be diagnosed at advanced stages according to the published studies; similarly, eight of our patients were diagnosed with T3 and T4 tumors. The lowest survival rate was determined in a 73-year-old patient with an ampullary MANEC, whereas the longest overall survival was 51 months in the patient with the gastric tumor. A limitation of our study may be the potential bias due to the fact that the study was conducted in a tertiary center designed for advanced gastrointestinal cancer surgery.

Despite the limited number of patients, this study is the most extended case series involving the Turkish population. MANECs are rare tumors diagnosed postoperatively using immunohistochemical evaluation. Although the presentation and surgical management are similar to that of pure adenocarcinomas or NECs, patients must undergo postoperative multidisciplinary oncologic and surgical management as soon as the tumor is diagnosed. Additional studies with more extended number of cases are required for determining the optimal treatment management and classification of these tumors.

## Figures and Tables

**Table 1:**
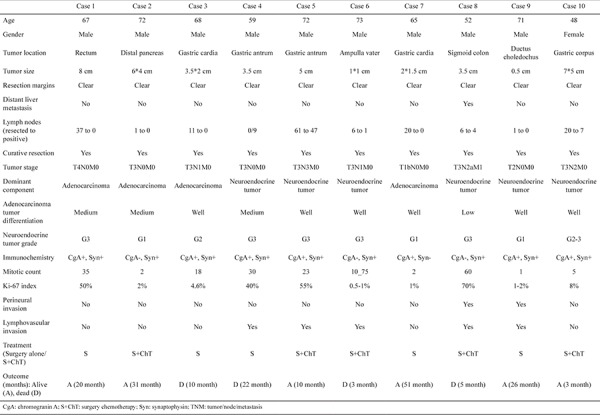
Demographics and tumor characteristics of participants
